# ESEA: Discovering the Dysregulated Pathways based on Edge Set Enrichment Analysis

**DOI:** 10.1038/srep13044

**Published:** 2015-08-12

**Authors:** Junwei Han, Xinrui Shi, Yunpeng Zhang, Yanjun Xu, Ying Jiang, Chunlong Zhang, Li Feng, Haixiu Yang, Desi Shang, Zeguo Sun, Fei Su, Chunquan Li, Xia Li

**Affiliations:** 1 College of Bioinformatics Science and Technology, Harbin Medical University, Harbin, 150081, PR China; 2 School of Medical Informatics, Daqing Campus, Harbin Medical University, Harbin, 150081, PR China; 3 College of Basic Medical Science, Heilongjiang University of Chinese Medicine, Harbin 150040, PR China

## Abstract

Pathway analyses are playing an increasingly important role in understanding biological mechanism, cellular function and disease states. Current pathway-identification methods generally focus on only the changes of gene expression levels; however, the biological relationships among genes are also the fundamental components of pathways, and the dysregulated relationships may also alter the pathway activities. We propose a powerful computational method, Edge Set Enrichment Analysis (ESEA), for the identification of dysregulated pathways. This provides a novel way of pathway analysis by investigating the changes of biological relationships of pathways in the context of gene expression data. Simulation studies illustrate the power and performance of ESEA under various simulated conditions. Using real datasets from p53 mutation, Type 2 diabetes and lung cancer, we validate effectiveness of ESEA in identifying dysregulated pathways. We further compare our results with five other pathway enrichment analysis methods. With these analyses, we show that ESEA is able to help uncover dysregulated biological pathways underlying complex traits and human diseases via specific use of the dysregulated biological relationships. We develop a freely available R-based tool of ESEA. Currently, ESEA can support pathway analysis of the seven public databases (KEGG; Reactome; Biocarta; NCI; SPIKE; HumanCyc; Panther).

The development of high-throughput experimental techniques such as microarray and next generation sequencing has led to amount of gene expression datasets. Thousands of dysregulated genes have been identified. To better understand the function of genes in the biological system, genes need to be studied in the context of the canonical biological pathways. The biological pathways analyses can help to insight into biological mechanism, cellular function and disease states^1^,^2^,^3^. Recently, a number of computational approaches have been developed to identify the dysregulated pathways associated with complex traits and human diseases^4^,^5^.

The classical enrichment analysis methods are developed by using the statistical models, such as Fisher’s exact test and hypergeometric test, to detect if the differentially-expressed genes are over- or under-represented in a predefined pathway^6^. A more sophisticated approach developed by Subramanian *et al.* is gene set enrichment analysis (GSEA)^7^. GSEA begins by ranking all genes according to their differential expression levels, and then uses weighted Kolmogorov-Smirnov statistic to measure if genes from a prespecified pathway are significantly overrepresented toward the top or bottom of the ranked gene list. Other similar strategies^8^,^9^,^10^ are also developed to identify the dysregulated pathways based on gene expression levels. As the measures of these methods are mainly based on investigating the alterations of gene expression levels, they can be deemed as node-centric methods. Although these methods make success in identifying dysregulated pathways, they do not directly consider the alterations of relationships among genes. Obviously, the relationships among genes, such as regulations among genes, are also the fundamental components of pathways, and their changes may play an important role in altering the activities of pathways^11^. The differential correlation analysis (e.g. differential coexpression) is able to identify the changes of relationships among genes^12^,^13^. Several approaches applied the differential correlation analysis to cancer gene expression datasets and found several regulations among genes involved in cancer with highly differential correlations, whereas their mean expression levels had hardly changed^14^,^15^,^16^. This illustrate that the changes of relationships among genes independently of gene expression levels, and would be extremely important to infer underlying biological insights.

Approaches based on the changes of relationships among genes (deemed as edge-centric methods) have been proposed to investigate dysregulated pathways. Zhang *et al.* proposes an interaction-based gene set analysis method (IB-GSA), which identifies enriched gene interaction (correlation) effects on a phenotype of interest in the framework of gene set analysis^17^. Gene set co-expression analysis (GSCA) calculates pairwise co-expressions for all gene pairs within a gene set, and introduces a dispersion index to quantify the difference of gene set between two biological conditions^18^. Liu *et al.* proposes gene interaction enrichment and network analysis (GIENA) to identify dysregulated pathways in complex diseases. GIENA defines several functions to model the biologically relevant gene interactions, and then identifies dysregulated interactions and pathways enriched in dysregulated interactions^11^. Although these methods identify some dysregulated pathways that are biologically meaningful, they generally regard the pathways as gene sets and do not take advantage of the inherent pathway structure information embedded in the pathways. In fact, pathways are models containing the structure information, such as interaction, regulation, modification, and binding etc. between genes, not simple sets of genes^1^,^2^,^3^. Exploiting the pathway structure in pathway identification analysis would improve our understanding of delicate pathway functions and the specificity of results^4^,^5^. However, the above edge-centric methods mainly identify dysregulated pathways by comparing the differential correlations for all gene pairs within the pathways, whereas ignoring pathway’s own structure information. Thus some differential-correlation relationships among genes identified in a pathway may result from other pathways.

Several recent methods effectively used pathway structure in identifying dysregulated pathways. ScorePage takes advantage of the shortest distances between genes in pathways for the analysis of changes in activity of metabolic pathways^19^. Tarca *et al.* proposes signaling pathway impact analysis (SPIA), which combines the positions and interactions of genes in the pathways with classical over-representation evidence in prioritizing risk signaling pathways^20^. Pathway enrichment analysis (PWEA) calculates a score, called “Topological Influence Factor (TIF)”, for each gene by using the shortest distances between genes in pathways, and then the degree of differential expression is weighted by their corresponding TIF to infer perturbed pathways^21^. Although these methods adopt the pathway structure information and achieve good results, they just use the pathway structure as evidences for connecting genes in pathways, whereas ignoring the changes of expression correlations between genes appearing in the pathway structure. These methods, which adopted pathway structure, actually use genes as entities, and thus belong to node-centric methods.

In this study, we developed a powerful edge-centric method, Edge Set Enrichment Analysis (ESEA), to identify dysregulated pathways by investigating the changes of inherent biological relationships embedded in pathways in the context of gene expression data. ESEA integrates pathway structure (e.g. interaction, regulation, modification, and binding etc. between genes) and differential correlation among genes. The biological pathways were collected from the seven public databases (KEGG^1^; Reactome^2^; Biocarta, www.biocarta.com; NCI/Nature Pathway Interaction Database^3^; SPIKE^22^; HumanCyc^23^; Panther^24^). We first converted each pathway in these databases into a graph with genes as nodes and biological relationships as edges. A background set of edges was constructed by extracting the edges from all the converted pathway graphs. We then applied an information-theoretic measure to quantify the change of correlation between genes in each edge based on gene expression data. An edge list was formed by ranking the edges according to their changes of correlation. Finally, we used the weighted Kolmogorov-Smirnov statistic to evaluate each pathway by mapping the edges in the pathway to the edge list. Using extensive simulation studies, we illustrated the power and performance of ESEA under various simulated conditions. We applied the ESEA method to p53 mutation, Type 2 diabetes and lung cancer datasets, and compare our results with five other pathway enrichment analysis methods. Based on these analyses, we validated that ESEA can produce biologically meaningful outcomes.

## Methods

**Figure 1:**
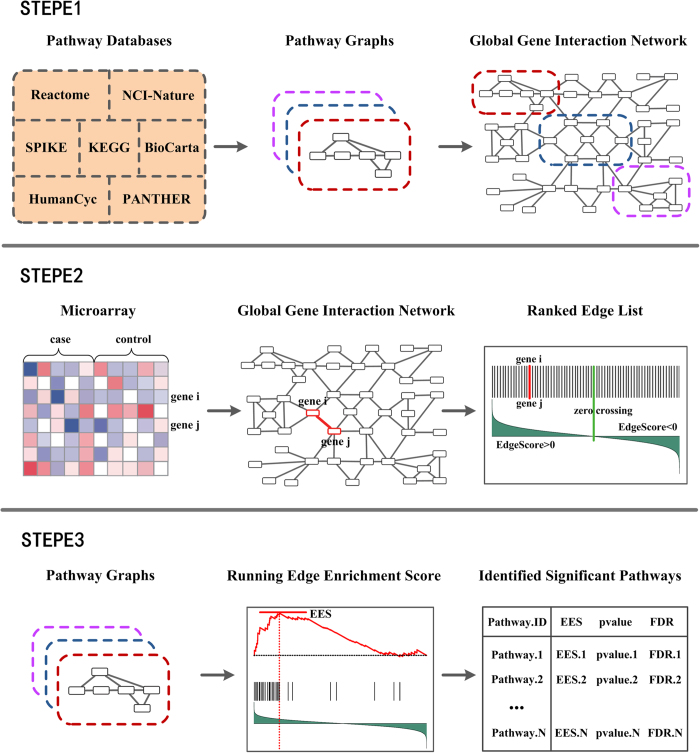
Flow diagram of the methodology. Step 1. Pathways in the seven pathway databases are converted to the corresponding pathway graphs. These pathway graphs are merged into a global gene interaction network, and all the edges in the global network are used as the background set of edges. Step 2. Gene expression data is mapped to the edges in the global network. The differential correlation score for each edge (EdgeScore) are estimated, and a ranked edge list is formed according to the EdgeScore. Step 3. Edges in a given pathway are mapped to the ranked edge list, and the edge enrichment score of pathway is calculated by walking down the list. The pathways are prioritized by FDR after permutation test.

ESEA was developed to identify dysregulated pathways based on the changes of biological relationships of pathways in the context of gene expression data. A flow diagram of the ESEA methodology is shown in Fig. 1. The main steps consist of (1) converting pathways into graphs and constructing the background set of edges based on the converted graphs; (2) estimating differential correlation scores of edges in the context of gene expression data; (3) calculating the edge enrichment score for each pathway in the pathway database. We have implemented ESEA as an R-based package, which is publicly available on CRAN (http://cran.r-project.org/web/packages/ESEA/).

### Dataset for analysis

We used three cases to illustrate the ESEA method. The first case was p53 mutation dataset published by Olivier *et al.*^25^. This dataset detected gene expression in response to the status of transcription factor p53, and comprised 50 samples of NCI-60 cell lines with 17 cell lines carrying native p53 status and 33 cell lines carrying mutated p53 status. The second case, obtained from Mootha *et al.*^26^, was diabetes dataset which investigated the transcriptional profiles of smooth muscle biopsies among patients with normal glucose tolerance (NGT), impaired glucose tolerance (IGT) and type 2 diabetes mellitus (DM2). Because our method focused on the binary comparison with the strongest disparity, we used the transcriptional profiles of NGT samples (17 subjects) and DM2 samples (17 subjects) in the study. The above two datasets were downloaded from the GSEA web set (http://www.broadinstitute.org/gsea/index.jsp). The third case was two independent lung cancer datasets (GSE7670 and GSE10072) published by Su *et al.*^27^ and Landi *et al.*^28^. These two gene expression datasets includes 54 (27 tumor and 27 normal tissues in GSE7670) and 107 (58 tumor and 49 normal tissues in GSE10072) samples respectively, and are available in the NCBI Gene Expression Omnibus (http://www.ncbi.nlm.nih.gov/geo).

### Constructing the background set of edges

We collected human pathways from the seven popular public databases (KEGG; Reactome; Biocarta; NCI; SPIKE; HumanCyc; Panther). There are more than 2300 pathways totally (Supplementary Table S1), which contain pathway structure information (e.g. interaction, regulation, modification, and binding etc. between genes). To extract the pathway structure information, we converted each pathway in the above databases into an undirected graph using the graphite software package^29^. Each node in the graph represents a gene, and each edge represents a relationship such as interaction, regulation or modification etc. between genes in the pathways. The edge set for each pathway can be extracted from the corresponding pathway graph. We then merged these pathway graphs into a global gene interaction network, which covers 8,894 nodes (genes) and 164,826 edges (interactions). All the edges in the global network, which correspond to the biological relationships of pathways, were used as the background set of edges. This background set can be obtained from our “ESEA” package (http://cran.r-project.org/web/packages/ESEA/).

### Differential correlation analysis for each edge

Differential correlation analysis was used to identify the changes of relationships among genes in the context of gene expression data. The information theoretic measure of statistical dependence, mutual information (MI), can estimate the correlation between the expression profiles of two genes^30^,^31^. The MI is always non-negative. If and only if two gene expression variables are statistically independent, the MI is zero.

We mapped the gene expression data to the background set of edges, and retained the edges in the background when both genes in the edge were mapped. For each edge, we estimated the MI between two genes in the edge using parmigene package, which gives more precise results with less computational costs^32^. The differential correlation score for an edge (*EdgeScore*) was defined as:


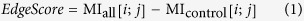


where MI_all_[*i*; *j*] represents the MI between the expression profiles of the two genes (*i* and *j*) in the edge across all samples; MI_control_[*i*; *j*] represents the MI between the expression profiles of the two genes (*i* and *j*) in the edge across control samples. According to the *EdgeScore*, each edge could be classified as either a gain of correlation (GoC), loss of correlation (LoC), or no change (NC). Specifically, we tested whether the MI increased (*EdgeScore* > 0) or decreased (*EdgeScore* < 0) when the samples with the specific phenotype were added to control samples. We defined the edge as GoC (LoC or NC) if the *EdgeScore* > 0 (<0 or =0), which refers to the correlation of the two genes in the edge is gained (lost or no change) in the specific phenotype. If an edge is strongly correlated with the specific phenotype, its *EdgeScore* will highly deviate from zero. We ranked the *N* edges in the background set to form a edge list *L* = {*e*_*1*_, *e*_*2*_, …*e*_*N*_} according to decreasing *EdgeScore*.

### Calculating the enrichment score of pathway

For each pathway in the seven pathway database (KEGG; Biocarta; Reactome; NCI; SPIKE; HumanCyc; Panther), the edge set can be extracted from the corresponding pathway graph. We therefore created the edge sets of pathways for each of the above databases, which can be obtained from our “ESEA” package (http://cran.r-project.org/web/packages/ESEA/). For a given database, pathways with more than 15 edges or less than 1000 edges in the expression dataset were used in the analysis. This will avoid overly narrow or broad functional pathways.

We mapped the edges in a predefined pathway to the ranked edge list *L* = {*e*_*1*_, *e*_*2*_, …*e*_*N*_}. If the edge set in this pathway significantly cluster at the top or bottom of the entire ranked list *L*, the pathway will be associated with the specific phenotype. We used the weighted Kolmogorov-Smirnov statistic to calculate an edge enrichment score (*EES*), which reflects the degree to which a pathway is overrepresented toward the extremes (top or bottom) of the edge list *L*. This statistic has been used in GSEA previously. But, it is used as statistic test of nodes. In the paper, we used it as statistic test of edges. Specifically, at a given position *i* in the list *L*, we evaluated the fraction of edges in the pathway (*F*_*InP*_) weighted by their *EdgeScore* and the fraction of edges not in the pathway (*F*_*NotP*_) as follows:


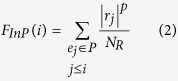



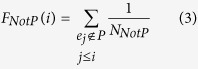


where *N*_*R*_ = 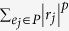; *r*_*j*_ is the *EdgeScore* of edge *j*; *N*_*NotP*_ represents the number of edges in the list *L* not in the pathway. The parameter *p* is used to weight the *EdgeScore* of the edges in the pathway, and we set *p* = 1 in the study. With the position *i* walking down the list *L*, the *EES* of the pathway (*EES*(*P*)) is calculated as the maximum deviation from zero of *F*_*InP*_ − *F*_*NotP*_. The *EES*(*P*) will be high if the edges in the pathway cluster at the top or bottom of the list, but if the edges randomly distributed at the list, the *EES*(*P*) will be small. According to the sign the *EES*(*P*), the pathways could be classified as GoC pathway (*EES*(*P*) > 0), LoC pathway (*EES*(*P*) < 0) and NC pathway (*EES*(*P*) = 0), which indicate the pathways are enriched by edges with GoC, LoC and NC respectively.

### Statistical significance analysis

To estimate the statistical significance (empirical *p-value*) of the *EES*(*P*), we performed a gene-based permutation test procedure that preserves the sample labels and gene expression data. Specifically, we permuted gene labels and recomputed the *EES*(*P*) for the permutated data. The background set of *EES* was generated after performing *N* permutations, and was designated as *EES*_*perm*_. When the observed *EES*(*P*) > 0, the *p-value* was computed as *p-value* = *M/N*, where *M* is the number of *EES*_*perm*_ greater than the observed *EES*(*P*); when the observed *EES*(*P*) < 0, *p-value* = *M/N*, where *M* is the number of *EES*_*perm*_ less than the observed *EES*(*P*). The permutation times *N* was set at 1000 for the examples in this study. Because of our method mainly studies the changes of correlation between genes, the gene-based permutation would be reasonable for identifying dysregulated pathway enriched by differential correlations relationships. To correct for multiple comparisons, we adjusted the empirical *p-values* by using false discovery rate (FDR) method proposed by Benjamini and Hochberg^33^. In the study, the FDR at 0.05 was used as pathway significance threshold.

Actually, only partial differential correlations relationships in a significant pathway will participate in the studied phenotype^12^,^17^. Thus, it is meaningful to extract the core member of edges in the significant pathway that contribute to the *EES*(*P*). Here, the core subset of edges in a significant pathway is defined as the edges appear in the ranked edge list *L* at and before (or after if *EES*(*P*) < 0) the point where *EES*(*P*) is obtained. The core subset of edges is expected to be more likely associated with the biological process of an interesting phenotype.

To account for the size of the pathway and allow inter-pathway comparisons with *EES*, we further normalized the observed *EES*(*P*). The normalized edge enrichment score (*NEES*) for each pathway was computed by:


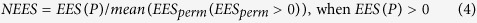


or


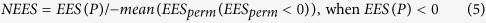


where *EES*_*perm*_(*EES*_*perm*_ > 0) or *EES*_*perm*_(*EES*_*perm*_ < 0) represent the vector of positive or negative scores in the *EES*_*perm*_ respectively.

### Generation of simulated data

To assess the performance of the ESEA approach, we performed simulated study. We simulated gene expression dataset with 2000 genes. A simulated background set of edges was constructed by selecting 10000 different genes pairs.

#### Simulating edge sets of pathways

We generated 100 pathways with edges, and the edges were extracted from simulated background set of edges. Only the first pathway was defined as causal pathway including differential correlation edges (risk edges). We chose 100 edges from simulated edge background set to generate this causal pathway. Thus, the maximum number of risk edge is 100. In real biological settings, only parts of edges in a risk pathway are differential correlative. We therefore introduced a parameter *γ*, the percentage of risk edge in the causal pathway, and we considered *γ* ∈{0.25, 0.50, 0.75, 1.00}. Other 99 pathways were simulated from null models, namely, none of the correlations of edges in these pathways were changed between two phenotypes of interest. The edges in these null pathways were chose from the remaining 9900 edges in simulated background set, and the sizes of these null pathways were randomly drawn from a uniform distribution U[50,150].

#### Simulating gene expression data

For gene expression dataset, we simulated 50 controls and 50 cases with 2000 genes from multivariate normal distribution 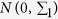 and 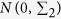 respectively. The matrix 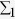 was set to an identity matrix of size 2000. 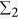 was set to a symmetric matrix of size 2000, and its elements are assigned by:


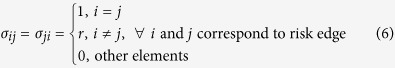


the parameter *r* controls the strength of correlation between genes in the risk edge. As the correlation between genes in the risk edge vary in strength, we consider *r* ∈{0.1, 0.2, …, 0.9}. A risk edge with larger *r* indicates that the edge possesses greater differential correlation degree between cases and controls. To ensure that 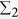 is positive definite, the elements of 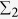 which correspond to risk edges were selected from different rows and different columns.

## Results

We illustrated the ESEA method using simulated data and real biological data. The simulation study was firstly conducted to assess the power and performance of ESEA in a fully controlled setting. We then explored the effectiveness of ESEA to provide biologically meaningful insights using two real expression datasets from p53 mutation and type 2 diabetes. In each case, we searched for significantly associated pathways from one or two of the seven public pathway databases (KEGG; Reactome; Biocarta; NCI; SPIKE; HumanCyc; Panther). We also provided a point of comparison by analyzing each data using GSEA. We then test the consistency of method by applying ESEA to two independent lung cancer datasets. Finally, we compared the results of ESEA with five other pathway enrichment analysis methods.

### Simulation study

We conducted extensive simulation studies to illustrate the power and performance of ESEA under various conditions. We simulated a gene expression data with 2000 genes and an edge background set with 10000 edges. 100 pathways were generated by extracting edges from simulated background set. Only the one of 100 defined pathways was used as casual pathway containing risk edges. The remaining 99 pathways were used as a null model, and the size of these null pathways were randomly drawn from a uniform distribution U[50,150]. Under real biological situations, not all edges in the casual pathway are differential correlative (risk), and the correlation between genes in the risk edge varies in strength. We thus introduced two parameters: *γ*, the percentage of risk edge in the causal pathway and *r*, the strength of correlation between genes in the risk edge, to test how exactly these parameters influence the power and performance of ESEA method.

We designed various scenarios in the simulated study by selecting different combination of *r*, *r* ∈{0.1, 0.2, …, 0.9} and *γ*, *γ* ∈{0.25, 0.50, 0.75, 1.00}. For each scenario, we performed 200 replicates and the power was calculated as the proportion of replicates for which the *p*-value for the causal pathway was less than 0.05. We plotted power curves by selecting each *γ* in {0.25, 0.50, 0.75, 1.00} and used *r* = {0.1, 0.2, …, 0.9} respectively (Supplementary Figure S1). At a given *γ*, such as *γ* = 0.50, the power curve rises with *r* increasing, and the curve with larger *γ* rises faster. In the case of the same *r*, a larger *γ* corresponds to a larger power. With *r* approximates to 0.9, the power curve of each test under different *γ* ∈{0.25, 0.50, 0.75, 1.00} approximates to 1 (Supplementary Figure S1).

**Figure 2:**
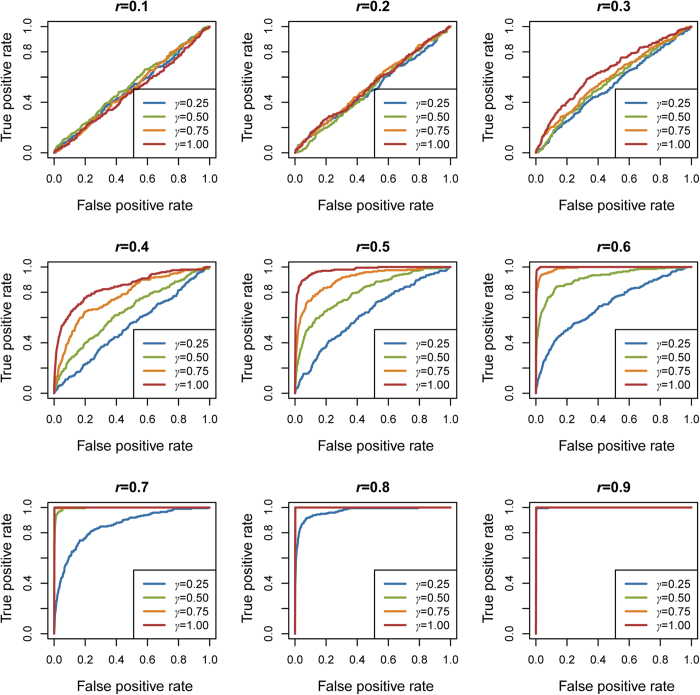
ROC curves of simulation studies. ROC curves of four *γ* levels (*γ* ∈{0.25, 0.50, 0.75, 1.00}) at different *r* levels (*r* ∈{0.1, 0.2, …, 0.9}) in the simulated studies.

We further used the receiver-operating characteristic (ROC) analysis to compare the performance of the algorithm under various scenarios (Fig. 2). The causal pathway and 99 null pathways were used as true positive set and true negative set respectively. When given *r* and *γ*, the ROC curve plots the true-positive rate (TPR) versus the false-positive rate (FPR) subject to the threshold (*p*-value) separating the identification results in 200 replicates. To compare different curves obtained by ROC analysis, we calculated the area under the ROC curve (AUC) for each curve (Supplementary Figure S2). When *r* ≤ 0.3 (the first row of Fig. 2), the ROC curves mix together, and the AUC for each curve is relatively small. When 0.3 < *r *≤ 0.6, the ROC curves separate according to *γ*, and a larger *γ* corresponded to a larger AUC. When *r* ≥ 0.7 (the third row of Fig. 2), almost all the AUCs exceed 0.9, indicating ESEA is able to identify the causal pathway with strong sensitivity and specificity. These results are actually what one would expect: the performance of ESEA method was influenced by both the strength of correlation between genes in the risk edge (*r*) and the percentage of risk edge (*γ*), and increased values for each or both of these two parameters would increase the power and performance of method.

### Analyses of p53 mutation data

Our first case was gene expression dataset of p53 status from the NCI-60 collection of cancer cell lines^25^. This dataset comprised 50 samples of NCI-60 cell lines with 17 cell lines carrying native p53 status and 33 cell lines carrying mutated p53 status. We mapped the expression data to the edges in background set, and this resulted in 74898 edges with the genes in them were mapped.

We first applied ESEA to identify KEGG pathways associated with p53 mutation. With FDR<0.05 pathway significance threshold, ESEA yielded five statistically significant pathways enriched by edges with gain of correlation (GoC pathways) (Table 1). The full list of ranked pathways was listed in the Supplementary Table S2. These significant pathways were all clearly reported to be associated with p53 mutation status. The most significant pathway was cysteine and methionine metabolism pathway. Benavides *et al.* demonstrated that methionine inhibited cellular growth dependent on the native p53 status of cancer cells, and this inhibited effects were loss in mutated p53 status^34^. The second significant pathway was alcoholism pathway, and the alcohol consumption have been proposed to be associated with p53 mutations in non-small cell lung cancer^35^. Xiong *et al.* demonstrated that the dilated cardiomyopathy caused by loss of Mdm4 (an inhibitor of the p53 tumor suppressor) was dependent on p53 dose^36^. An important role of p53 has been revealed in regulating interactions of cells with the ECM and participating in the interpretation of ECM-derived signalling cues^37^. Moreover, the colorectal cancer pathway presents two major mechanisms of genomic instability. Rodrigues *et al.* concluded that mutation of the p53 gene was one of the commonest genetic alterations in the progression of human colorectal cancer^38^.

To provide a comparison analysis, we also applied GSEA to p53 mutation dataset to identify KEGG pathways. With the default threshold of method (FDR < 0.25), GSEA identified one significant pathway: N-Glycan biosynthesis. Although this pathway may be associated with the p53 function, ESEA exclusively identified five statistically significant pathways associated with the p53 function.

**Figure 3:**
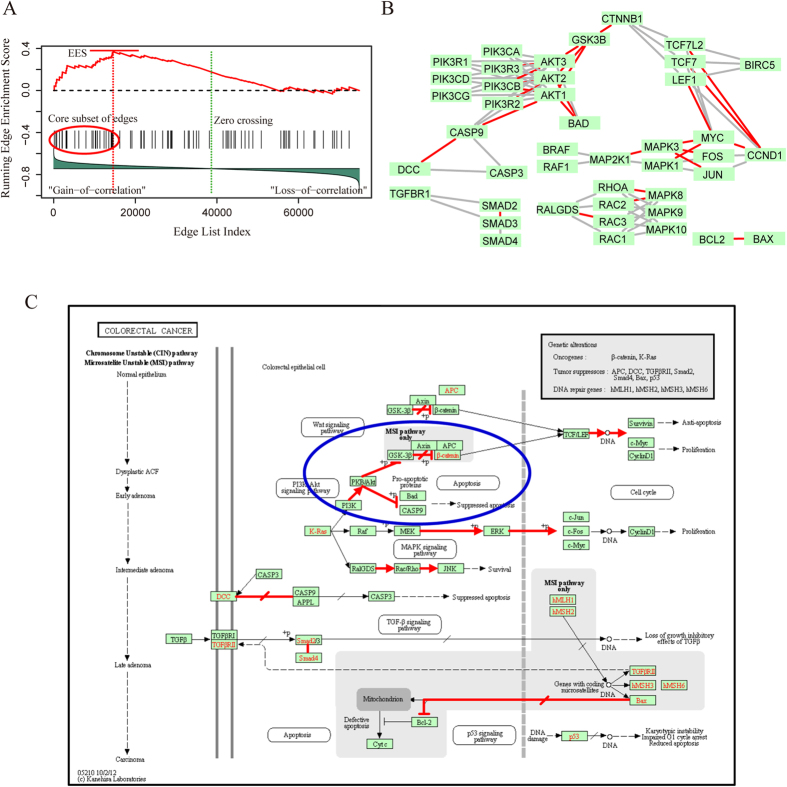
Running enrichment score and annotating core subset of edges to the colorectal cancer pathway. (**A**) Running-sum statistic is calculated by walking down the edge list, and the maximum deviation from zero of the statistic is used as edge enrichment score of the pathway. (**B**) Core subset of edges are extracted and mapped to the pathway graph. (**C**) Colorectal cancer pathway in KEGG, and the biological relationships which correspond to the core edges are annotated with red.

We further explain the rationale of ESEA method in the colorectal cancer pathway. Specifically, the edges in the converted pathway graph were mapped to a ranked edge list, and 80 edges were obtained (Fig. 3A). As the edge list was ranked based on the *EdgeScore* representing differential correlation degree, the edges locate close to the top or bottom of the list may tend to be dysregulated. The accumulation of multiple dysregulated edges may result in the pathway dysregulated. To reflect the degree to which the edges in the pathway cluster toward the extremes (top or bottom) of the edge list, the edge enrichment score of the pathway (*EES*(*P*)) was calculated by walking down the edge list. A running-sum statistic was calculated by increasing it when we encounter an edge in the pathway and decreasing it when we encounter edges not in the pathway (Fig. 3A). The maximum deviation from zero of the statistic was used as *EES*(*P*) (The detail information for each edge in the pathway was listed in the Supplementary Table S3). The top 27 edges in the pathway, which contributed to the *EES*(*P*), were defined as core subset of edges. These core edges were mapped to the pathway graph. A series of dysregulated relationships were found (Fig. 3B).

These dysregulated relationships were then mapped to the original pathway, and a region of PI3K/AKT and β-catenin signaling cascade was identified (blue circle in Fig. 3C). Some evidences were found in the literatures for the biological significance of this signaling cascade. In benign cells, p53 inhibits the PI3K/AKT signaling through the transcriptional activation of phosphatase and tensin homolog (PTEN)^39^. And the p53 mutation may activate this PI3K/AKT signaling, which has been demonstrated to be correlated with cancer cell growth and survival^40^,^41^. Interestingly, the core edges “AKT2|PIK3R2”, “AKT2|PIK3CB” and “AKT3|PIK3R3” etc. which correspond to this PI3K/AKT signaling cascade were identified to be gain of correlation (GoC) in p53 mutation samples. In addition, the expression of native p53 would inhibit the β-catenin in human cells through the serine/threonine kinase glycogen synthase kinase 3β (GSK-3β)-mediated phosphorylation^42^,^43^. The p53 mutation status would disorder the inhibitory effect of GSK-3β on β-catenin, and this would trigger the accumulation of β-catenin which has been proposed to be associated with colorectal cancer^43^. We also found that the core edge “CTNNB1|GSK3B” corresponding to the relationship between β-catenin and GSK-3β was assigned with GoC. These observations showed that ESEA can found a strong connection between colorectal cancer pathway and p53 mutation.

Secondly, we applied ESEA and GSEA to identify Biocarta pathways associated with p53 mutation. With FDR < 0.05, ESEA identified one statistically significant GoC pathway: CDK regulation of DNA replication (The full lists of ranked pathways was listed in the Supplementary Table S4). GSEA identified three significant pathways with the default threshold of method (FDR < 0.25), including hypoxia and p53 in the cardiovascular system, BCR signaling pathway and nerve growth factor pathway. Although GSEA found more significant pathways, ESEA is able to find something new dysregulated pathway, CDK regulation of DNA replication, which has been demonstrated to be associated with p53 function^44^. Specifically, activation of p53 by DNA damage may lead to enhanced Cdc6 destruction, which is triggered by inhibition of CDK2-mediated Cdc6 phosphorylation at serine 54. The destruction of Cdc6 may block initiation of DNA replication. Conversely, loss of p53 function may lead to stabilization of Cdc6, whose effect may produce more replicating cells^44^.

### Analyses of type 2 diabetes data

Our second case we chose to evaluate was type 2 diabetes data published by Mootha *et al.*^26^. This dataset investigated the transcriptional profiles of smooth muscle biopsies among patients with normal glucose tolerance (NGT), impaired glucose tolerance (IGT) and type 2 diabetes mellitus (DM2). Because of ESEA focused on the binary comparison with the strongest differential correlation between genes, we used the transcriptional profiles of smooth muscle biopsies of 17 NGT and 17 DM2 samples in this case. We mapped the expression data to the background set of edges, and thus obtained 97375 edges with the genes in them were mapped.

We applied ESEA and GSEA to identify Reactome pathways associated with type 2 diabetes respectively. With FDR < 0.05, ESEA identified seven statistically significant pathways, including three GoC pathways and four LoC pathways (Table 2). The full list of ranked pathways was listed in the Supplementary Table S5. GSEA identified four statistically significant pathways with the default threshold of method (FDR < 0.25). Surprisingly, the significant pathways in ESEA and GSEA did not share any overlap. Although the pathways found by GSEA may be associated with type 2 diabetes, the pathways found by ESEA are also reported to be implicated in the progression of type 2 diabetes. For instance, downregulation of ERBB2/ERBB3 signaling pathway was proposed to play an important role in maintaining insulin signaling, and the dysregulated of this pathway may causes an impairment of insulin action which is closely related to type 2 diabetes^45^. Chaperonin-mediated protein folding is critical for the survival and proper function of cells, and impaired protein folding has been implicated in type 2 diabetes^46^. Peptide ligand-binding receptors have been reported to be important drug targets for the treatment of type 2 diabetes^47^,^48^. Excessive and inappropriate activation of NFkB and MAP kinases may contribute to insulin resistance and type 2 diabetes^49^,^50^. These results indicate that ESEA may complement the GSEA in identifying dysregulated pathways.

**Figure 4:**
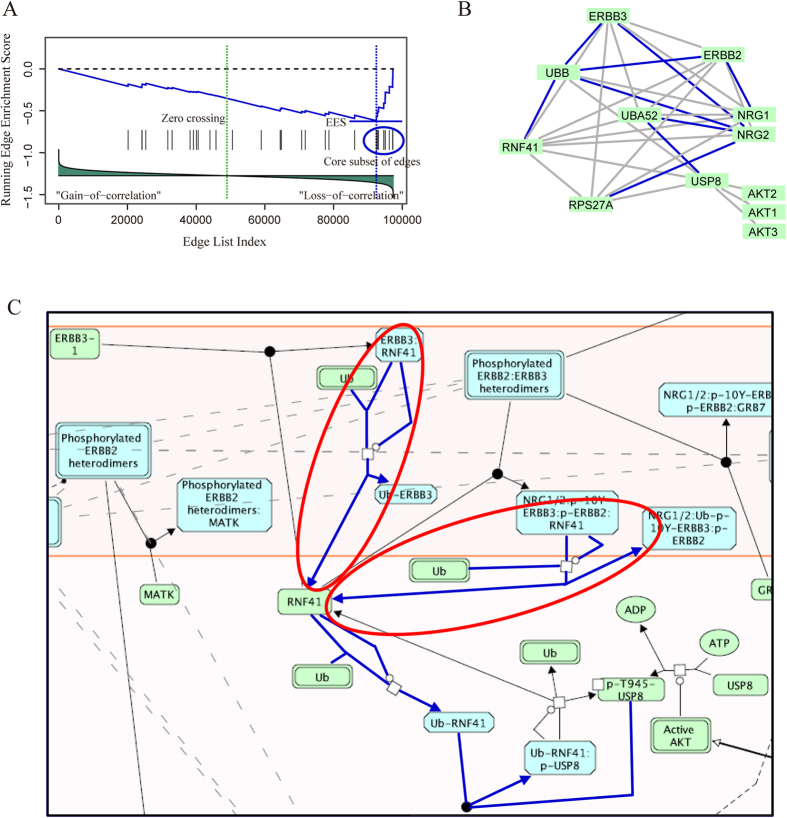
Running enrichment score and annotating core subset of edges to the downregulation of ERBB2/ERBB3 signaling pathway. (**A**) Running-sum statistic is calculated by walking down the edge list, and the maximum deviation from zero of the statistic is used as edge enrichment score of the pathway. (**B**) Core subset of edges are extracted and mapped to the pathway graph. (**C**) Downregulation of ERBB2/ERBB3 signaling pathway in Reactome, and the biological relationships which correspond to the core edges are annotated with blue.

The downregulation of ERBB2/ERBB3 signaling pathway was used as an example to illustrated how it was identified by ESEA. The edges in this pathway were mapped to the ranked edge list, and a running-sum statistic was calculated by walking down the list (Fig. 4A). The core subset of edges were extracted and mapped to the pathway graph (Supplementary Table S6 and Fig. 4B), and then mapped to the original pathway (Fig. 4C). In this pathway, most of the biological relationships, corresponding to the core edges, were demonstrated to be associated with the initiation and progression of type 2 diabetes. Two major actions, including E3 ubiquitin ligase (RNF41) ubiquitinates inactive ERBB3 and activated ERBB2/ERBB3 (red circles in Fig. 4C), degrade and regulate ERBB2/ERBB3 level in the cell^51^. Loss of these actions may cause the accumulation of ERBB2/ERBB3 level, which may impair insulin action associated with the development of type 2 diabetes^45^. Interestingly, the core edges “ERBB3|UBB”, “RNF41|UBB” and “ERBB2|UBB” etc. (Supplementary Table S6), which correspond to the above actions were identified to be loss of correlation in type 2 diabetes samples. These observations indicate that ESEA is able to found dysregulated pathways affected by dysfunctional biological relationships.

### Analyses of two lung cancer data

**Figure 5:**
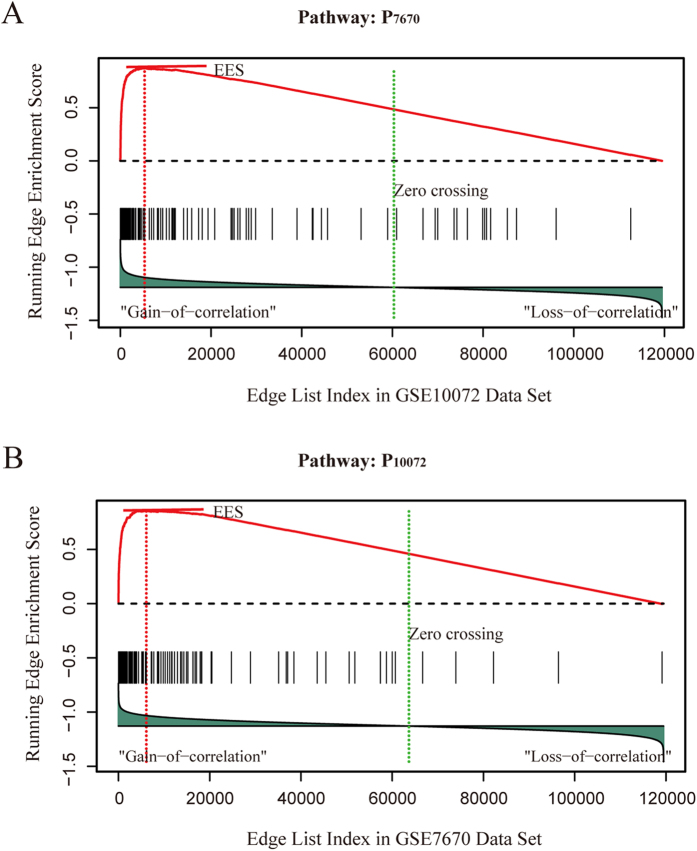
Enrichment plots for edges with gain of correlation across lung cancer studies. (**A**) Enrichment plots for the pathway *P*_7670_ against the GSE10072 datasets. (**B**) Enrichment plots for the pathway *P*_10072_ against the GSE7670 datasets.

To test if the ESEA method could obtain consistent results across different datasets, we used two independent derived lung cancer datasets (GSE7670 and GSE10072) for analysis. We defined two edge sets of pathways, *P*_7670_ and *P*_10072_, to be the top 200 edges with gain of correlation in the GSE7670 and GSE10072 datasets respectively. To reveal if ESEA can obtain the similarity between the GSE7670 and GSE10072 datasets, we firstly mapped the pathway *P*_7670_ to the entire ranked edge list from the GSE10072 dataset (Fig. 5A). The pathway *P*_7670_ shows a strong significant enrichment in the GSE10072 data (*NEES* = 4.14, *p-value* < 0.001). We then mapped the pathway *P*_10072_ to the entire ranked edge list from the GSE7670 dataset (Fig. 5B), and the pathway *P*_10072_ is significant enriched in the GSE7670 data (*NEES* = 4.02, *p-value* < 0.001). These results indicate that the ESEA method is able to detect strong consistent signal between independently derived lung cancer datasets.

We further explored whether ESEA could provide consistent pathways in lung cancer. We performed ESEA on the two lung cancer datasets with the Reactome pathways. To provide a more general comparison, the top 20 pathways from each lung dataset were used to test how many pathways were overlapped. Interestingly, approximately half of the pathways (9 pathways) were shared between the two studies across the top 20 pathways (Table 3). These overlapped pathways were clearly related to the three key biological functions: DNA replication, cell cycle and extracellular matrix organization, which are associated with cell growth and proliferation. Moreover, almost all of these overlapped pathways have been reported to be directly or indirectly related to the initiation and progression of lung cancer. Specifically, activation of the pre-replicative complex has been proposed to be correlated with lung cancer development^52^. Zheng *et al.* reported that dysregulated G2/M checkpoint function was associated with an increased risk of lung cancer^53^. Polo-like kinase gene expression could provide an independent prognostic indicator for patients with non-small cell lung cancer^54^.

### Comparison of ESEA with other methods

To explore whether ESEA could provide new biological insights in identifying important pathways, we applied DAVID^6^, GSEA^7^, SPIA^20^, PWEA^21^ and PathNet^55^ to identify dysregulated KEGG pathways in the p53 mutation dataset and Type 2 diabetes dataset. With the default threshold for each method, 13 statistically significant pathways were identified by all the above methods in the p53 mutation dataset (Supplementary Table S7). In detail, the classical methods such as DAVID and GSEA identified one and two significant pathways respectively (DAVID’s FDR < 0.05 and GSEA’s FDR < 0.25). The improved methods such as SPIA, PWEA and PathNet found two, one and four significant pathways respectively (SPIA’s FDR < 0.05, PWEA’s FDR < 0.01 and PathNet’s FWER < 0.05). With FDR < 0.05, ESEA identified five statistically significant pathways. Through comparing the results of these methods, we found that the overlaps of the significant pathways among all the above methods are very few. This indicates that these methods are complementary. Interestingly, we found that ESEA identified five statistically significant pathways, which were simultaneously missed by other methods (Supplementary Table S7). The significant pathways in ESEA, such as the cysteine and methionine metabolism, ECM-receptor interaction, colorectal cancer pathway etc., have been well reported to be associated with p53 mutation state^34^,^37^,^38^. The reason for the difference results between ESEA and other methods may be because the ESEA and other methods use different strategies to identify dysregulated pathways. The ESEA method uses the differentially correlation relationships between genes to identify dysregulated pathways, and other methods mainly use the differentially expressed genes. Similarly, in the Type 2 diabetes dataset, ESEA identified five statistically significant pathways which were simultaneously missed by the other methods (Supplementary Table S8). These results indicate that the ESEA method may uncover something new dysregulated pathways.

## Discussion

Identifying dysregulated canonical biological pathways can help us to understand biological mechanism, cellular function and disease states. According to the entities used by the pathway identification methods, these methods can be naturally classified as node-centric (gene based) and edge-centric (gene-gene relationships based) methods. The recent pathway identification methods mainly belong to node-centric methods (e.g. GSEA), which focus on investigating the changes of gene expression levels between cases and controls. Although these node-centric methods achieved good results, they did not consider the changes of relationships among genes which may also alter the activities of pathways. Some edge-centric methods were thus developed to detect the changes of relationships among genes in identifying dysregulated pathways. However, they mainly compared the difference for all gene pairs within the pathways and did not take advantage of the inherent pathway structure (e.g. interaction, regulation, modification, and binding etc.). Actually, pathways are models describing the pathway structure, not simple sets of genes. Thus, in these methods, some differential-correlation relationships identified in a pathway may result from other pathways. ESEA was developed as an edge-centric method by integrating pathway structure and differential correlation among genes, which may improve the specificity of results in identifying the dysregulated pathways.

Because the prior pathway structure of the recent pathway databases was generally incomplete; we thus collected more than 2300 human pathways from the seven popular pathway databases (Supplementary Table S1) to construct the background set of edge. Nevertheless, the background may still incomplete. With the update and accumulation of the pathway databases, the background set of edges would be increasingly more complete, which will continue to increase the power of ESEA. To reflect the specific disease processes information, we mapped the gene expression data with cases and controls to the edge background. The mutual information (MI) can provide a better and more general criterion to investigate relationships between variables^30^,^31^. We thus used the MI to estimate the differential correlation score (*EdgeScore*) between the expression profiles of two genes in the edge. According to the *EdgeScore*, the edges were classified as either a gain of correlation (GoC), loss of correlation (LoC), or no change (NC). This could provide more delicate information for the biological relationships in the development of complex diseases.

In the study, ESEA is designed to identify the dysregulated pathways by investigating the changes of inherent biological relationships (e.g. interaction, regulation, modification, and binding etc. between genes) embedded in pathways in the context of gene expression data. This means that the dysregulated pathways identified by ESEA are enriched by the specific dysfunctional biological relationships between genes. The strategy of ESEA is different from the recent pathway enrichment analysis methods (e.g. DAVID, GSEA, SPIA, etc.), which identify the dysregulated pathways based on the differentially expressed genes. To explore whether ESEA could provide new biological insight in identifying important pathways, we further applied DAVID^6^, GSEA^7^, SPIA^20^, PWEA^21^ and PathNet^55^ to identify dysregulated pathways in the p53 mutation dataset and Type 2 diabetes dataset. By comparing the results of ESEA with five other methods in the p53 mutation dataset, we found that ESEA identified five statistically significant pathways, which were simultaneously missed by other methods (Supplementary Table S7). The significant pathways in ESEA, such as the cysteine and methionine metabolism, ECM-receptor interaction, colorectal cancer pathway etc., have been well reported to be associated with p53 mutation state^34^,^37^,^38^. Similarly, in the Type 2 diabetes dataset, ESEA identified five statistically significant pathways which were simultaneously missed by the other methods (Supplementary Table S8). Our results indicate that ESEA may uncover something new dysregulated pathways, and thus may complement other pathway enrichment analysis methods.

By detecting the dysregulated pathways obtained from ESEA, we found that these pathways were enriched by the dysregulated biological relationships. For the colorectal cancer pathway identified in p53 mutation data, 27 core edges, such as “AKT2|PIK3R2”, “AKT2|PIK3CB” and “AKT3|PIK3R3” etc., were found. Through mapping these core edges to the original pathway, a region of PI3K/AKT and β-catenin signaling cascade (blue circle in Fig. 3C) associated with p53 function was effectively identified^39^,^40^,^41^,^42^. For the downregulation of ERBB2/ERBB3 signaling pathway identified in type 2 diabetes data, nine core edges, such as “ERBB3|UBB”, “RNF41|UBB” and “ERBB2|UBB” etc. were found, and two major pathway actions (red circles in Fig. 4C) associated with type 2 diabetes were identified^45^,^51^. These results indicate that the ESEA method is able to find the delicate and specific results, and thus may provide underlying biological insights into complex traits and human diseases.

In order to make the EAEA to be broadly applicable, we have implemented ESEA as a flexible R-based package, which is freely available on CRAN (http://cran.r-project.org/web/packages/ESEA/). The users input interesting gene expression data with case and control samples, and the dysregulated pathways can then be inferred. The edge sets of pathways have been created for each of the seven pathway databases (KEGG; Reactome; Biocarta; NCI; SPIKE; HumanCyc; Panther). ESEA can be flexibly applied to the pathways in a given databases. The ESEA method was applied to gene expression microarrays in the study, and it can also be apply to the transcriptome profiling from next-generation sequencing (RNA-Seq).

## Additional Information

**How to cite this article**: Han, J. *et al.* ESEA: Discovering the Dysregulated Pathways based on Edge Set Enrichment Analysis. *Sci. Rep.* **5**, 13044; doi: 10.1038/srep13044 (2015).

## Supplementary Material

Supplementary Information

Supplementary Table S2

Supplementary Table S4

Supplementary Table S5

## Figures and Tables

**Table 1:** KEGG pathways identified by ESEA with FDR < 0.05 in the p53 dataset.

Pathway	Size of edge	NEES	FDR	Character
Cysteine and methionine metabolism	66	1.75	<0.001	GoC
Alcoholism	590	1.50	<0.001	GoC
Dilated cardiomyopathy	319	1.44	<0.001	GoC
ECM-receptor interaction	466	1.35	<0.001	GoC
Colorectal cancer	80	1.60	0.04	GoC

**Table 2:** Reactome pathways identified by ESEA with FDR < 0.05 in the type 2 diabetes dataset.

Pathway	Size of edge	NEES	FDR	Character
Downregulation of ERBB2-ERBB3 signaling	31	−2.11	<0.001	LoC
Amino acid and oligopeptide SLC transporters	131	−1.73	<0.001	LoC
Chaperonin-mediated protein folding	152	−1.64	<0.001	LoC
Protein folding	161	−1.59	<0.001	LoC
Peptide ligand-binding receptors	79	1.57	<0.001	GoC
TRAF6 mediated induction of NFkB and MAP kinases upon TLR7-8 or 9 activation	227	1.42	<0.001	GoC
Nucleosome assembly	524	1.31	<0.001	GoC

**Table 3:** Overlapped Reactome pathways between the two lung cancer studies across the top 20 pathways.

Pathway	Biological Functions	GSE10072		GSE7670	
		NEES	FDR	NEES	FDR
Activation of the pre-replicative complex	DNA replication, cell cycle	3.36	<0.001	2.11	<0.001
DNA strand elongation	DNA replication, cell cycle	3.09	<0.001	2.44	<0.001
G2-M Checkpoints	cell cycle	3.04	<0.001	2.21	<0.001
Unwinding of DNA	DNA replication, cell cycle	3.03	<0.001	2.82	<0.001
Activation of ATR in response to replication stress	cell cycle	3.03	<0.001	2.17	<0.001
Condensation of Prometaphase Chromosomes	cell cycle	2.63	<0.001	2.42	<0.001
Polo-like kinase mediated events	cell cycle	2.45	<0.001	2.13	<0.001
Cyclin A-B1 associated events during G2-M transition	cell cycle	2.43	<0.001	2.13	<0.001
Collagen biosynthesis and modifying enzymes	extracellular matrix organization	2.22	<0.001	2.00	<0.001
