# Supplementary material for: ESEA: Discovering the Dysregulated Pathways based on Edge Set Enrichment Analysis
Source: Sci Rep. 2015 Aug 12;5:13044. doi: 10.1038/srep13044 (PMC4533315; doi:10.1038/srep13044)
Supplement: Supplementary Information [file srep13044-s1.pdf]

## Supplementary Information

# ESEA: Discovering the Dysregulated Pathways based on Edge Set Enrichment Analysis

Junwei Han[1,†], Xinrui Shi[1,†], Yunpeng Zhang[1,†], Yanjun Xu[1], Ying Jiang[3], Chunlong Zhang[1], Li Feng[1], Haixiu Yang[1], Desi Shang[1], Zeguo Sun[1], Fei Su[1], Chunquan Li[2,]* and Xia Li[1,]*

[1] College of Bioinformatics Science and Technology, Harbin Medical University, Harbin, 150081, PR China

[2] School of Medical Informatics, Daqing Campus, Harbin Medical University, Harbin, 150081, PR China

[3] College of Basic Medical Science, Heilongjiang University of Chinese Medicine, Harbin 150040, PR China

*Correspondence and requests for materials should be addressed to C.L.( lcqbio@163.com) or X.L.( lixia@hrbmu.edu.cn)

[†]These authors contributed equally to this work

**Inventory of Supplementary Information**

1. Supplementary Figure S1
2. Supplementary Figure S2
3. Supplementary Table S1
4. Supplementary Table S3
5. Supplementary Table S6
6. Supplementary Table S7
7. Supplementary Table S8

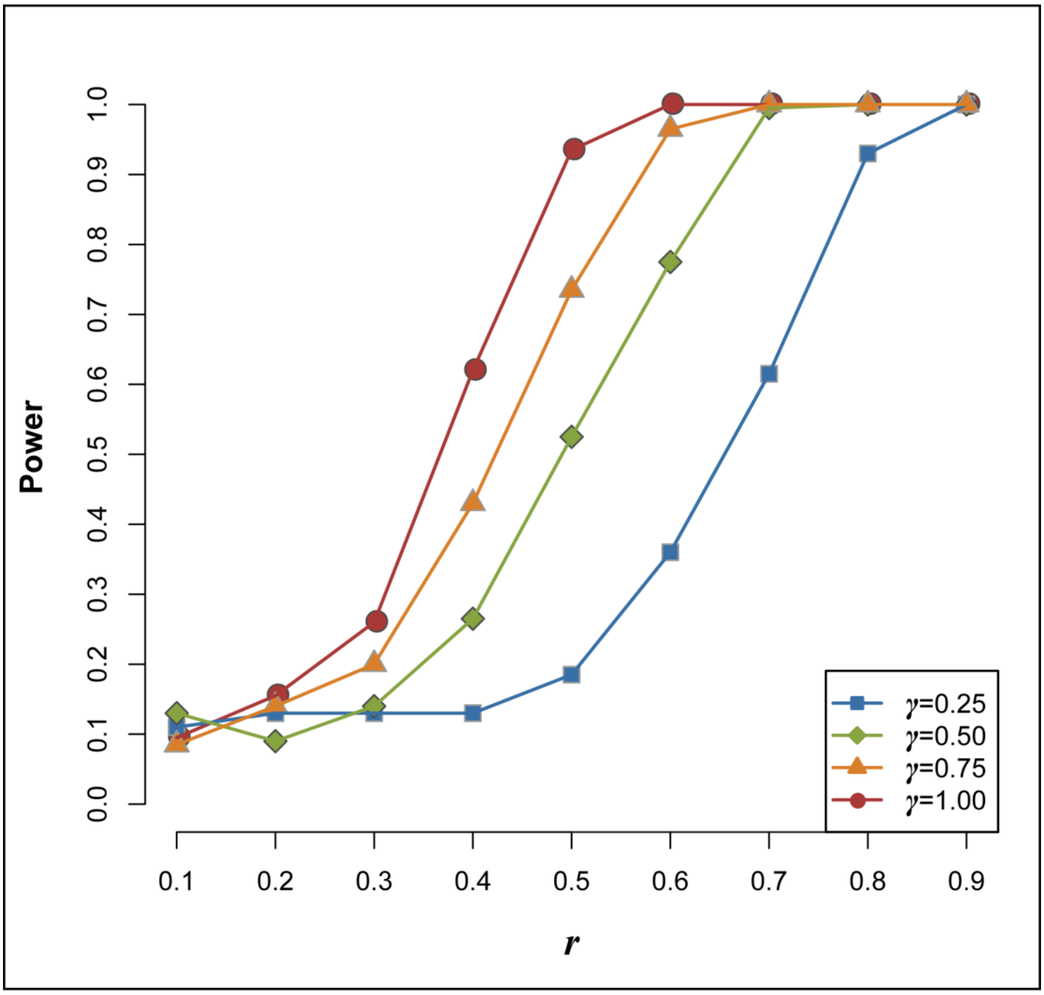


**Figure S1.** Power curves of simulation studies. Power curves of four $\gamma$ levels ($\gamma \in \{0.25, 0.50, 0.75, 1.00\}$) at different $r$ levels ($r \in \{0.1, 0.2, \ldots, 0.9\}$) in the simulated studies.

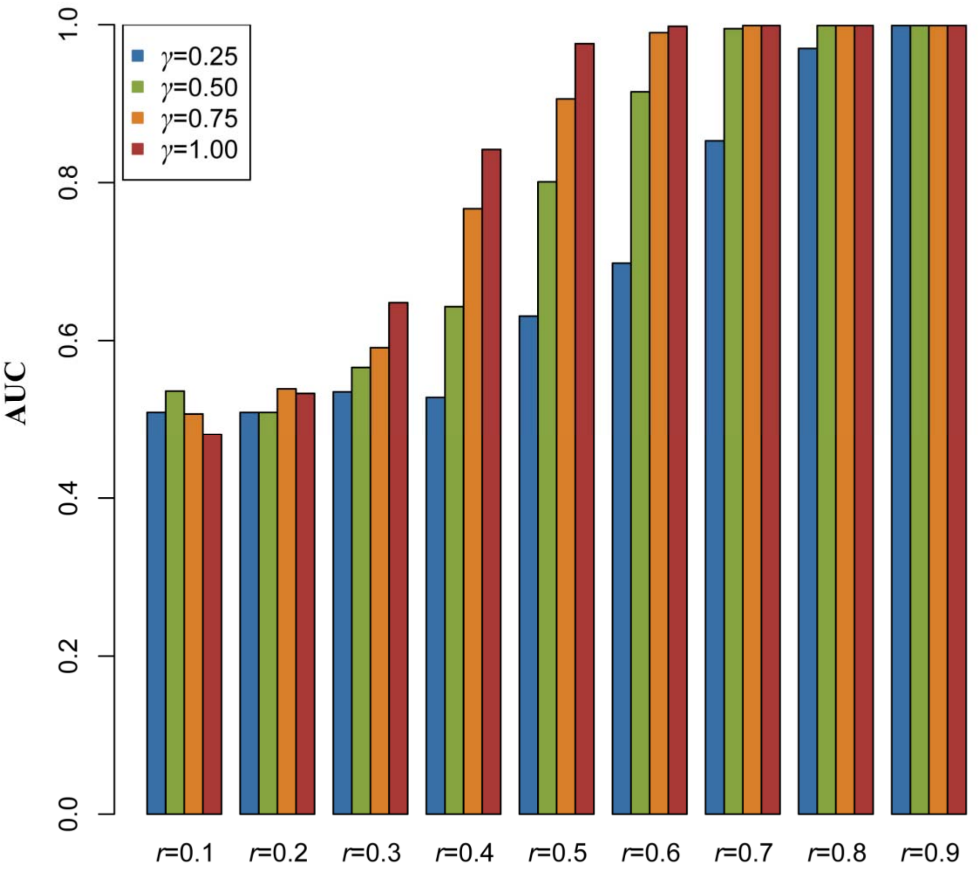


**Figure S2.** Bar chart of Area Under ROC Curve (AUC) for four γ levels (γ∈{0.25, 0.50, 0.75, 1.00}) at different r levels (r∈{0.1, 0.2,…, 0.9}) in the simulated studies.

## Table S1. Overview of pathways in the seven databases

| Pathway Database | Pathway Number | Gene Number | Interaction Number |
|---|---|---|---|
| KEGG | 236 | 4694 | 47588 |
| Reactome | 1347 | 6787 | 104606 |
| Biocarta | 247 | 1036 | 5424 |
| NCI | 212 | 2400 | 14246 |
| HumanCyc | 174 | 600 | 1471 |
| SPIKE | 28 | 798 | 1521 |
| Panther | 84 | 1113 | 12148 |
| Total | 2328 | 8894 | 164826 |

**Table S3. Detail information for each edge in the colorectal cancer pathway**

| # | EdgeID | List Loc | EdgeScore | REES | CORE_ENRICHMENT |
|---|---|---|---|---|---|
| 1 | MAPK8\|RHOA | 113 | 0.431 | 0.0434 | YES |
| 2 | AKT1\|CASP9 | 518 | 0.351 | 0.0745 | YES |
| 3 | RAC3\|RALGDS | 726 | 0.333 | 0.106 | YES |
| 4 | MAPK3\|MYC | 1392 | 0.296 | 0.128 | YES |
| 5 | AKT2\|BAD | 2018 | 0.273 | 0.148 | YES |
| 6 | AKT2\|GSK3B | 2098 | 0.27 | 0.175 | YES |
| 7 | AKT2\|PIK3R2 | 3003 | 0.248 | 0.189 | YES |
| 8 | AKT2\|PIK3CB | 3142 | 0.245 | 0.213 | YES |
| 9 | CTNNB1\|GSK3B | 3256 | 0.243 | 0.236 | YES |
| 10 | MAPK1\|MYC | 5180 | 0.211 | 0.233 | YES |
| 11 | CCND1\|TCF7L2 | 6138 | 0.199 | 0.241 | YES |
| 12 | AKT1\|GSK3B | 7899 | 0.179 | 0.236 | YES |
| 13 | BAX\|BCL2 | 9373 | 0.165 | 0.233 | YES |
| 14 | LEF1\|MYC | 9654 | 0.163 | 0.246 | YES |
| 15 | AKT1\|BAD | 10190 | 0.158 | 0.256 | YES |
| 16 | AKT3\|PIK3CB | 10330 | 0.157 | 0.27 | YES |
| 17 | MAP2K1\|MAPK3 | 10710 | 0.154 | 0.281 | YES |
| 18 | SMAD2\|SMAD4 | 11311 | 0.148 | 0.289 | YES |
| 19 | CCND1\|MYC | 11312 | 0.148 | 0.304 | YES |
| 20 | JUN\|MAPK3 | 12320 | 0.14 | 0.305 | YES |
| 21 | AKT3\|PIK3R3 | 12822 | 0.137 | 0.313 | YES |
| 22 | MAPK8\|RAC2 | 13503 | 0.132 | 0.317 | YES |
| 23 | AKT1\|PIK3CB | 14071 | 0.128 | 0.323 | YES |
| 24 | FOS\|MAPK3 | 14235 | 0.127 | 0.334 | YES |
| 25 | AKT3\|GSK3B | 14342 | 0.126 | 0.346 | YES |
| 26 | CASP9\|DCC | 14357 | 0.126 | 0.359 | YES |
| 27 | CCND1\|TCF7 | 14559 | 0.125 | 0.369 | YES |
| 28 | CASP3\|DCC | 16189 | 0.114 | 0.359 | NO |
| 29 | AKT2\|PIK3R1 | 18839 | 0.0984 | 0.334 | NO |
| 30 | BIRC5\|LEF1 | 19163 | 0.0966 | 0.34 | NO |
| 31 | BIRC5\|TCF7 | 21347 | 0.0843 | 0.319 | NO |
| 32 | AKT2\|PIK3CD | 21495 | 0.0835 | 0.326 | NO |
| 33 | AKT3\|PIK3CA | 22079 | 0.0805 | 0.327 | NO |
| 34 | AKT3\|BAD | 22183 | 0.08 | 0.334 | NO |
| 35 | MAPK9\|RAC3 | 24116 | 0.0698 | 0.315 | NO |
| 36 | AKT2\|CASP9 | 24868 | 0.0662 | 0.312 | NO |
| 37 | MAPK9\|RAC2 | 26715 | 0.0568 | 0.293 | NO |
| 38 | CTNNB1\|LEF1 | 27908 | 0.0512 | 0.282 | NO |
| 39 | AKT1\|PIK3CA | 28106 | 0.0502 | 0.285 | NO |
| 40 | MAPK10\|RAC2 | 28657 | 0.0476 | 0.283 | NO |
| 41 | MAP2K1\|RAF1 | 28856 | 0.0467 | 0.285 | NO |

| | | | | | |
|---|---|---|---|---|---|
| 42 | AKT3\|CASP9 | 29020 | 0.0459 | 0.287 | NO |
| 43 | SMAD3\|TGFBR1 | 32055 | 0.0314 | 0.25 | NO |
| 44 | MYC\|TCF7 | 32966 | 0.027 | 0.241 | NO |
| 45 | MAPK9\|RAC1 | 33192 | 0.0259 | 0.241 | NO |
| 46 | CASP3\|CASP9 | 33212 | 0.0258 | 0.243 | NO |
| 47 | MAPK10\|RAC3 | 34249 | 0.021 | 0.231 | NO |
| 48 | AKT3\|PIK3R2 | 34980 | 0.0175 | 0.223 | NO |
| 49 | SMAD3\|SMAD4 | 35554 | 0.0148 | 0.217 | NO |
| 50 | AKT1\|PIK3CD | 38148 | 0.00249 | 0.183 | NO |
| 51 | AKT1\|PIK3R2 | 41509 | -0.014 | 0.139 | NO |
| 52 | AKT3\|PIK3CG | 42250 | -0.0175 | 0.131 | NO |
| 53 | MYC\|TCF7L2 | 42568 | -0.0191 | 0.129 | NO |
| 54 | JUN\|MAPK1 | 42915 | -0.0208 | 0.127 | NO |
| 55 | RALGDS\|RHOA | 43468 | -0.0234 | 0.122 | NO |
| 56 | MAPK10\|RHOA | 43514 | -0.0236 | 0.124 | NO |
| 57 | AKT1\|PIK3R3 | 44189 | -0.027 | 0.117 | NO |
| 58 | MAPK8\|RAC3 | 44541 | -0.0289 | 0.116 | NO |
| 59 | RAC1\|RALGDS | 46004 | -0.036 | 0.0999 | NO |
| 60 | CTNNB1\|TCF7 | 46898 | -0.0406 | 0.0922 | NO |
| 61 | AKT3\|PIK3CD | 47836 | -0.0457 | 0.0844 | NO |
| 62 | SMAD2\|TGFBR1 | 48817 | -0.0508 | 0.0766 | NO |
| 63 | CCND1\|LEF1 | 50876 | -0.062 | 0.0555 | NO |
| 64 | AKT2\|PIK3CG | 55672 | -0.0898 | 0.000774 | NO |
| 65 | MAPK9\|RHOA | 56361 | -0.094 | 0.00136 | NO |
| 66 | CTNNB1\|TCF7L2 | 56375 | -0.0941 | 0.011 | NO |
| 67 | FOS\|MAPK1 | 56616 | -0.0957 | 0.0177 | NO |
| 68 | CCND1\|FOS | 56976 | -0.0981 | 0.0231 | NO |
| 69 | BIRC5\|TCF7L2 | 57556 | -0.102 | 0.026 | NO |
| 70 | AKT1\|PIK3CG | 57846 | -0.104 | 0.0329 | NO |
| 71 | BRAF\|MAP2K1 | 59757 | -0.117 | 0.0195 | NO |
| 72 | MAPK8\|RAC1 | 61344 | -0.129 | 0.0117 | NO |
| 73 | MAPK10\|RAC1 | 63787 | -0.15 | -0.00533 | NO |
| 74 | RAC2\|RALGDS | 64036 | -0.152 | 0.00719 | NO |
| 75 | CCND1\|JUN | 66113 | -0.174 | -0.00245 | NO |
| 76 | AKT3\|PIK3R1 | 68374 | -0.202 | -0.0117 | NO |
| 77 | AKT1\|PIK3R1 | 68810 | -0.208 | 0.00419 | NO |
| 78 | MAP2K1\|MAPK1 | 69404 | -0.217 | 0.0189 | NO |
| 79 | AKT2\|PIK3R3 | 70557 | -0.239 | 0.0283 | NO |
| 80 | AKT2\|PIK3CA | 72425 | -0.286 | 0.0331 | NO |

**Table S6. Detail information for each edge in the downregulation of ERBB2/ERBB3 signaling pathway**

| # | EdgeID | List Loc | EdgeScore | REES | CORE_ENRICHMENT |
|---|---|---|---|---|---|
| 1 | NRG2\|UBA52 | 97262 | -0.498 | 0.00116 | YES |
| 2 | ERBB3\|NRG2 | 97204 | -0.474 | -0.112 | YES |
| 3 | ERBB2\|UBB | 96233 | -0.35 | -0.21 | YES |
| 4 | ERBB2\|NRG1 | 95060 | -0.297 | -0.277 | YES |
| 5 | UBA52\|USP8 | 95006 | -0.296 | -0.344 | YES |
| 6 | ERBB3\|UBB | 94591 | -0.284 | -0.407 | YES |
| 7 | NRG2\|RPS27A | 93079 | -0.252 | -0.456 | YES |
| 8 | NRG2\|UBB | 92779 | -0.247 | -0.511 | YES |
| 9 | RNF41\|UBB | 92450 | -0.241 | -0.564 | YES |
| 10 | ERBB3\|UBA52 | 86140 | -0.17 | -0.554 | NO |
| 11 | ERBB2\|UBA52 | 78647 | -0.122 | -0.515 | NO |
| 12 | NRG1\|UBA52 | 77586 | -0.116 | -0.532 | NO |
| 13 | ERBB2\|NRG2 | 71766 | -0.0877 | -0.499 | NO |
| 14 | RNF41\|USP8 | 70705 | -0.0828 | -0.508 | NO |
| 15 | ERBB3\|RNF41 | 64830 | -0.0581 | -0.466 | NO |
| 16 | NRG1\|RNF41 | 64508 | -0.0568 | -0.476 | NO |
| 17 | AKT2\|USP8 | 64462 | -0.0567 | -0.489 | NO |
| 18 | ERBB2\|ERBB3 | 58939 | -0.0359 | -0.445 | NO |
| 19 | NRG1\|UBB | 50588 | -0.00569 | -0.367 | NO |
| 20 | AKT3\|USP8 | 45773 | 0.011 | -0.319 | NO |
| 21 | RNF41\|UBA52 | 44005 | 0.017 | -0.303 | NO |
| 22 | NRG2\|RNF41 | 40577 | 0.0287 | -0.272 | NO |
| 23 | ERBB2\|RNF41 | 40099 | 0.0306 | -0.274 | NO |
| 24 | RNF41\|RPS27A | 39210 | 0.0337 | -0.272 | NO |
| 25 | ERBB3\|RPS27A | 38213 | 0.0373 | -0.269 | NO |
| 26 | NRG1\|RPS27A | 33015 | 0.057 | -0.224 | NO |
| 27 | AKT1\|USP8 | 31753 | 0.0619 | -0.224 | NO |
| 28 | UBB\|USP8 | 25363 | 0.0882 | -0.173 | NO |
| 29 | ERBB2\|RPS27A | 24262 | 0.0931 | -0.181 | NO |
| 30 | ERBB3\|NRG1 | 24210 | 0.0934 | -0.202 | NO |
| 31 | RPS27A\|USP8 | 20198 | 0.112 | -0.182 | NO |

**Table S7 Pathways identified by six methods (DAVID, GSEA, SPIA, PWEA, PathNet, ESEA) in the p53 mutation dataset.**

| PathwayName | David | GSEA | SPIA | PWEA | PathNet | ESEA |
|---|---|---|---|---|---|---|
| Cysteine and methionine metabolism | | | | | | √ |
| Alcoholism | | | | | | √ |
| Dilated cardiomyopathy | | | | | | √ |
| ECM-receptor interaction | | | | | | √ |
| Colorectal cancer | | | | | | √ |
| Type I diabetes mellitus | √ | | √ | | | |
| Graft-versus-host disease | √ | | √ | | | |
| N-Glycan biosynthesis | | √ | | | | |
| Folate biosynthesis | | | | √ | | |
| Parkinsons disease | | | | | √ | |
| PPAR signaling pathway | | | | | √ | |
| Ribosome | | | | | √ | |
| Alzheimers disease | | | | | √ | |

**Table S8 Pathways identified by six methods (DAVID, GSEA, SPIA, PWEA, PathNet, ESEA) in the Type 2 diabetes dataset.**

| PathwayName | David | GSEA | SPIA | PWEA | PathNet | ESEA |
|---|---|---|---|---|---|---|
| Circadian entrainment | | | | | | √ |
| Metabolism of xenobiotics by cytochrome P450 | | | | | | √ |
| Retinol metabolism | | | | | | √ |
| Retrograde endocannabinoid signaling | | | | | | √ |
| Terpenoid backbone biosynthesis | | | | | | √ |
| Alzheimer's disease | | √ | | | | |
| Huntington's disease | | √ | | | | |
| Nucleotide excision repair | | √ | | | | |
| Oxidative phosphorylation | | √ | | | | |
| Parkinson's disease | | √ | | | | |
| Ribosome | | √ | | | √ | |
| Huntingtons disease | | | | | √ | |
| Parkinsons disease | | | | | √ | |
